# Structural Variation Evolution at the 15q11-q13 Disease-Associated Locus

**DOI:** 10.3390/ijms242115818

**Published:** 2023-10-31

**Authors:** Annalisa Paparella, Alberto L’Abbate, Donato Palmisano, Gerardina Chirico, David Porubsky, Claudia R. Catacchio, Mario Ventura, Evan E. Eichler, Flavia A. M. Maggiolini, Francesca Antonacci

**Affiliations:** 1 Department of Biosciences, Biotechnology and Environment, University of Bari “Aldo Moro”, 70125 Bari, Italy; 2 Institute of Biomembranes, Bioenergetics, and Molecular Biotechnology (IBIOM), 70125 Bari, Italy; 3 Department of Genome Sciences, University of Washington School of Medicine, Seattle, WA 98195, USA; 4 Howard Hughes Medical Institute (HHMI), University of Washington, Seattle, WA 98195, USA; 5 Research Centre for Viticulture and Enology, Council for Agricultural Research and Economics (CREA), 70010 Bari, Italy

**Keywords:** segmental duplications, evolution, copy number variants, inversions, core duplicons

## Abstract

The impact of segmental duplications on human evolution and disease is only just starting to unfold, thanks to advancements in sequencing technologies that allow for their discovery and precise genotyping. The 15q11-q13 locus is a hotspot of recurrent copy number variation associated with Prader–Willi/Angelman syndromes, developmental delay, autism, and epilepsy and is mediated by complex segmental duplications, many of which arose recently during evolution. To gain insight into the instability of this region, we characterized its architecture in human and nonhuman primates, reconstructing the evolutionary history of five different inversions that rearranged the region in different species primarily by accumulation of segmental duplications. Comparative analysis of human and nonhuman primate duplication structures suggests a human-specific gain of directly oriented duplications in the regions flanking the *GOLGA* cores and *HERC* segmental duplications, representing potential genomic drivers for the human-specific expansions. The increasing complexity of segmental duplication organization over the course of evolution underlies its association with human susceptibility to recurrent disease-associated rearrangements.

## 1. Introduction

Our current knowledge of the extent of genetic variation shows that structural changes—insertions, duplications, deletions, and inversions of chromosomal segments—are extremely common in the human population [1,2,3,4]. Central to an understanding of structural variation is the interspersed segmental duplication (SD) architecture of human and ape genomes. Many structural variations are not random events but occur as a result of unequal crossovers between SDs, which act as catalysts for genomic instability with profound consequences in evolution and human disease [1,4,5,6,7,8,9,10,11,12,13,14,15,16,17].

Human chromosome 15 is particularly enriched in interspersed duplication blocks [18]. Specifically, within the 15q11-13 locus, a region spanning approximately 13 Mbp, there are five clusters of SDs (BP1, BP2, BP3, BP4 and BP5) ranging in size from ~400 kbp to 3 Mbp. These duplications define the breakpoints (BPs) of pathogenic copy number variants (CNVs) [19,20,21,22,23] and of human inversions, potentially causing predisposition to morbid CNV formation [8,24,25]. Recurrent CNVs at this locus are associated with Prader–Willi/Angelman syndromes (PWS/AS), developmental delay, autism, and epilepsy [26,27,28,29,30,31,32]. The *GOLGA* core duplicons have been previously shown to represent the focal point for the expansion and duplicative transposition of SDs [33], as well as the preferential sites of disease and evolutionary instability on chromosome 15 [8,10]. *GOLGA* belongs to a rapidly evolving gene family that has been subjected to a burst of SDs and to structural reorganization over the last 20 million years [34,35]. The duplication architecture around the *GOLGA* gene copies in human and great apes is complex and not yet well resolved since it is highly enriched for gaps and assembly errors even within the most recent versions of primate reference genomes [36]. Furthermore, within the common BP regions of PWS/AS, a chromosome 15 duplication containing *HERC2* has been identified [37]. These duplications mediated the transposition of four genes, which are considered candidate modulators of the PWS/AS phenotypes and may contribute to other disorders mapping to this specific region in humans [38]. Recent advances in long-read genome sequencing and assembly methods have enabled for the first time the production of truly complete telomere-to-telomere (T2T) assemblies of complex genomes [18,39], offering the opportunity to investigate the evolutionary differences in duplicated and structurally diverse regions of the genome between humans and the other apes.

Given the importance of the 15q11-q13 locus and the central role of its SDs in disease, we explored the architecture of this region in human and nonhuman primate (NHP) genomes using a combination of molecular cytogenetics, single-cell strand sequencing (Strand-seq), and near-complete genomic sequencing data with the aim to track evolutionary changes in the locus and better understand the mechanisms leading to genomic instability in evolutionary and disease contexts. Our results reveal patterns of structural variation and evolutionary differences in SD organization at the 15q11-q13 locus between humans and other apes that underlie human susceptibility to de novo recurrent rearrangements and disease.

## 2. Results

### 2.1. Strand-Seq Analysis of Primate Genomes

In order to reconstruct the evolutionary history of the regions flanked by the five SD blocks (BP1, BP2, BP3, BP4, and BP5) (Table S1), we compared the organization of the 15q11-q13 locus in human and NHPs. We took advantage of the Strand-seq data available for chimpanzee (Dorien), gorilla (GGO9), orangutan (PPY10), and macaque (MMU1) [11,40] (Figure S1a). Strand-seq allows the visualization of inversions by identifying DNA sequence strand switches (visualized in orange), indicating the inverted sequence with respect to the flanking regions in direct orientation (teal) [12,41,42]. Strand-seq analysis within the 13 Mbp at the 15q11-q13 locus (chr15:17,691,439-30,429,130—reference T2T CHM13v2.0/hs1) suggests a complex pattern of structural rearrangements in the investigated species. The chimpanzee sample shows a complete switch in orientation for the BP2-BP3 region, gorilla a partial switch for BP3-BP4 (indicative of a heterozygous inversion) and a complete switch in orientation for BP4-BP5, orangutan a complete switch in orientation from BP1 to BP4, and macaque for the whole region (from BP1 to BP5) (Figure S1a). The presence of SD mapping within the strand-switched regions suggests several possible inversion scenarios that cannot be resolved using Strand-seq only.

### 2.2. Evolutionary Origin of the Inversions

To achieve a more precise resolution of the 15q11-q13 region organization in both human and NHPs, we integrated the Strand-seq data with cytogenetic analyses. We developed interphase FISH assays to test each potential inversion on five chimpanzees, four gorillas, five orangutans, three macaques, and one marmoset (used as outgroup when necessary). We also evaluated the complete genome assemblies of our region of interest (chr15:17,691,439-30,429,130) for chimpanzee, gorilla, and orangutan (Data Availability Statement, Section 4) as well as the reference sequence for macaque (Mmul_10/rheMac10) in order to resolve the complex structural rearrangements that occurred in each of the analyzed species and precisely delineate the evolutionary history of the whole locus. The ape sequencing data and assemblies were generated by combining HiFi PacBio sequencing and ultra-long Oxford Nanopore Technologies data and have been made available as early-access to the genomics community as a resource (https://genomeark.github.io/t2t-all/, (accessed on 31 May 2023)) for the purpose of investigating individual regions and loci. A detailed description of the results obtained for each region of interest is provided below.

***BP1-BP2 analysis.*** The smallest region analyzed is BP1-BP2, which is only 300 kbp in size. Due to the size detection limits of molecular cytogenetics, the BP1-BP2 region was only analyzed by generating sequence homology plots (minimiro) between each primate and the CHM13 reference (T2T CHM13v2.0/hs1) (Figure S2). Sequence analysis shows that only orangutan and macaque are inverted compared to human, suggesting that the BP1-BP2 inversion likely occurred in the African great ape ancestor (Table 1 and Table S2).

***BP2-BP3 analysis.*** The BP2-BP3 region corresponds to the PWS/AS type II critical region and has been previously found to be inverted in the parents of AS patients [24]. To gain insight into the instability associated with disease at the BP2-BP3 region, we sought to characterize its organization in more detail in humans. Using a FISH assay in interphase nuclei, we tested 13 unrelated HapMap individuals for the presence of the ~4.7 Mbp BP2-BP3 inversion (Table S3). Out of 13 individuals, 12 were in direct orientation while only one sample (HG02492) of Punjabi ancestry, previously described to be inverted by Porubsky and colleagues [25], was heterozygous for the inversion (Table S3, Figure S3). By integrating our data with published Strand-seq data on 38 human individuals (which included four samples also tested by FISH) (Table S3) [25] and with data from Gimelli and collogues [24], we calculated the inverted allele frequency to be 2.8%.

**Figure 1:**
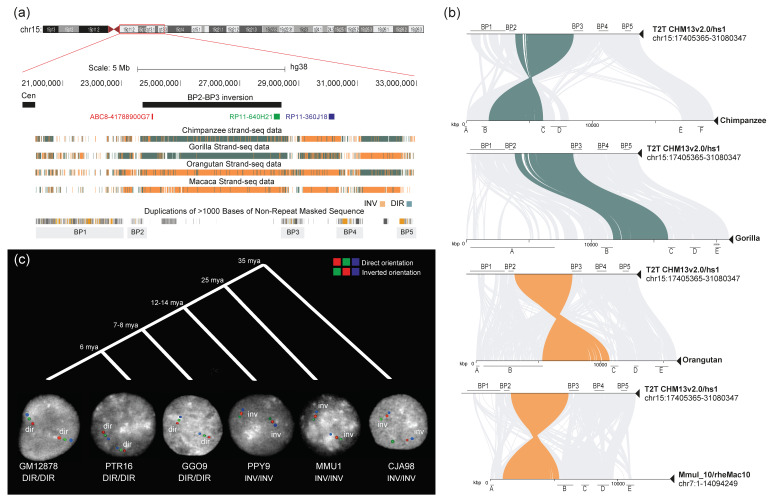
**BP2-BP3 inversion analysis.** (**a**) UCSC Genome Browser view of the BP2-BP3 region in humans. The black bar represents the putative inversion; fosmid and BAC clones used for FISH experiments on interphase nuclei are indicated by red, green, and blue bars. Strand-seq data for chimpanzee, gorilla, orangutan, and macaque are reported, showing a direct orientation of the region for chimpanzee and gorilla and an inverted orientation for orangutan and macaque. (**b**) Minimiro sequence homology plots between humans and nonhuman primates (NHPs) for the 15q11-q13 region are depicted. Teal and orange lines connect the BP2-BP3 orthologous regions between humans and NHPs, in direct and inverted orientation, respectively. The remaining orthologous regions of the 15q11-13 locus are connected using gray lines. (**c**) FISH results on interphase nuclei for the BP2-BP3 inversion are shown for each analyzed species. The color order indicates probes’ relative orientation, with red–green–blue signals showing the direct orientation and green–red–blue signals showing inverted haplotypes. FISH analyses show that orangutan, macaque, and marmoset (outgroup) are all inverted when compared to the human reference genome orientation, while chimpanzee and gorilla are direct. The timing of species divergences is also shown at the top (mya = million years ago). GM12878 = *Homo sapiens*; PTR = *Pan troglodytes*; GGO = *Gorilla gorilla*; PPY = *Pongo pygmaeus*; MMU = *Macaca mulatta*; CJA = *Callithrix jacchus*.

Strand-seq and minimiro sequence homology plots for primate genomes show that the BP2-BP3 region is inverted in chimpanzee, orangutan, and macaque with respect to the human reference genome (Figure 1a,b). However, chimpanzee has a *Pan*-specific well-known pericentric inversion [43,44] between the centromere and BP4; thus, BP2-BP3 appears inverted but is actually direct within the larger *Pan*-specific inversion of 6 Mbp (Figure S1a,b). To prevent any confusion, in all the following figures, we adjusted the coloring of the chimpanzee Strand-seq reads. Specifically, we switched the color assignment from the human centromere to BP4, in consideration of the *Pan*-specific pericentric inversion.

Using an interphase FISH assay on the NHP samples, we found that all the chimpanzees (n = 5) and gorillas (n = 3) are in direct orientation for the region, while all the orangutan (n = 5) and macaque individuals (n = 3) carry the inverted haplotype (Table S2, Figure 1c). Of note, while performing FISH hybridizations, we identified a heterozygous deletion in PPY10 overlapping with human fosmid probe ABC8-41788900G7 (red) (Table S4). The Strand-seq data for the same individual confirm a decrease in read depth for a ~600kbp region (Figure S4). Thus, for this individual, we were not able to assess the state of the BP2-BP3 region using FISH; however, based on the Strand-seq data for the region (with the exception of the deleted portion), we assume that PPY10 is homozygous for the inverted orientation like the other three orangutans tested. Taking into account the *Pan*-specific inversion, all our results suggest that the inversion occurred in the African great ape ancestor and is still polymorphic in humans (Table 1, Table S2 and Figure 1c).

***BP3-BP5 analysis.*** Strand-seq data alone are not sufficient to understand whether the BP3-BP5 region is inverted as a whole or whether two separate inversions (BP3-BP4 and BP4-BP5) occurred. The minimiro sequence homology plots suggest that for all the species, with the exception of macaque, there are two independent inversions (Figure S5). In order to confirm these results, we first tested the whole ~3.3 Mbp region using FISH in our NHP samples with the exception of chimpanzee, where the region is interrupted by the *Pan*-specific pericentric inversion. The FISH experiment results showed a direct orientation of the region for gorilla and orangutan, while macaque and marmoset (outgroup) were inverted. These data together show that this large inversion between BP3 and BP5 occurred in the great ape ancestor (Table 1, Table S2 and Figure S6).

***BP3-BP4 and BP4-BP5 analyses.*** Since the regions between BP3 and BP4 and between BP4 and BP5 seem to have harbored independent evolutionary inversions, we tested them individually using FISH. FISH experiments on the BP3-BP4 region showed an inverted orientation for chimpanzee and orangutan, while gorilla is polymorphic with an inverted allele frequency of 12.5%. Macaque and marmoset (the outgroup) instead carry the direct orientation. All the FISH results were confirmed using the minimiro sequence homology plots (Figure S5). Based on these results, the BP3-BP4 inversion likely occurred in the great ape ancestor with the human and gorilla orientations being either a result of incomplete lineage sorting or recurrent events (Figure S7, Table S2). The BP4-BP5 inversion has been extensively analyzed by Antonacci and colleagues [8]. Here, we performed additional experiments in order to increase the cohort of the individuals and extend the analysis to macaque and marmoset (the outgroup). Our results show that gorilla and marmoset have an inverted orientation, human (GM12878) is heterozygous for the inversions, while all the other NHPs tested carry the direct haplotype. We confirmed that marmoset carries the ancestral configuration using UCSC net alignment data for mouse [45,46,47]. All FISH results were confirmed using in silico sequence homology analysis. We conclude that the inversion occurred in the Catharrini ancestor with the human and gorilla configurations representing recurrent events as previously described [25] (Table 1, Table S2 and Figure S8).

### 2.3. Segmental Duplication Organization in Human

**Figure 2:**
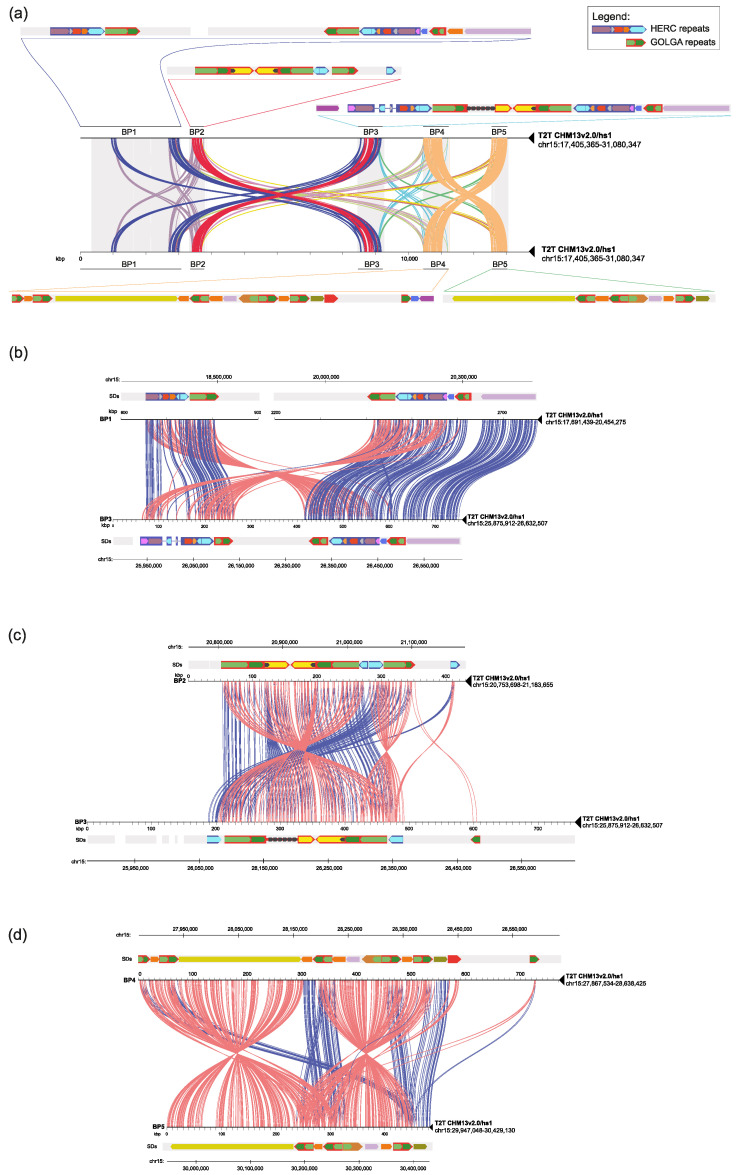
Human sequence homology plots of the 15q11-13 region. (**a**) Minimiro comparison of the whole 15q11-13 human locus against itself (T2T CHM13v2.0/hs1). The five SD blocks involved in pathogenic rearrangements are depicted (BP1 to BP5). Colored lines connect paralogous SDs between different BPs. A detailed map of the SDs’ organization and their relative orientation is depicted for each BP. Larger arrows indicate duplication modules containing core duplicons widespread along the locus. (**b**) Minimiro comparison of BP1 versus BP3 highlights homologous SDs between them. Red and blue lines represent sequences showing a relative inverted and direct orientation between the two BPs, respectively. (**c**) Minimiro comparison of BP2 versus BP3 highlighting homologous SDs between the two BPs. Red lines represent sequences showing a relative inverted orientation between the two BPs, and blue lines a direct orientation. (**d**) Minimiro comparison of BP4 versus BP5 highlighting homologous SDs between the two BPs. Red lines represent sequences showing a relative inverted orientation between BP4 and BP5, and blue lines a direct orientation.

Given the central role of SDs both in inversion onset during evolution and in pathogenic CNV predisposition, we analyzed the human SD organization at the 15q11-q13 locus looking for those that can putatively predispose to these rearrangements. To do this, we took advantage of the complete human reference genome [39] and we performed a minimiro comparison between the five human BPs. We reconstructed the detailed SD structure of the human direct haplotype (Figure 2a), improving the data already reported by Makoff and Flomen [21] and by Antonacci and colleagues [8]. Our pairwise analysis between SD blocks shows that the largest paralogous duplications identified are those mapping within the three BPs involved in human microdeletions, i.e., BP1-BP3, BP2-BP3, and BP4-BP5 (Figure 2). On the contrary, the other BPs do not have relevant homologous duplications between them (Figure S9). Our data highlight the presence, between BP1 and BP3, of a ~160 kbp duplication block with 99% similarity in both direct and inverted orientation (red and blue large arrows in Figure 2b) and a ~320 kbp block with 98% similarity in direct orientation (red/blue/light blue/red/pink arrows in Figure 2b). BP2 and BP3 comparison shows the presence of a ~100 kbp block with 98% similarity in both direct and inverted orientation (large red arrow in Figure 2c). Finally, a 35 kbp duplication with 99% similarity is present in both direct and inverted orientation between BP4 and BP5 (green repeats within the large red arrow in Figure 2d).

We assessed the genomic content of the SD blocks using the RepeatMasker tool (Combined Database: Dfam 3.0, rmblastn version 2.14.1+). This analysis revealed the presence of interspersed repeats, constituting approximately 41% to 47% of the sequence length for each block. Notably, the most prevalent classes of repetitive elements within these SD blocks were LINEs and SINEs, with the LINE1 and Alu elements being particularly prominent. These were followed by LTRs (Table S5). It is well documented that LINEs can play a structural role in promoting gene duplication [48], and Alu-mediated mechanisms have been suggested to enhance the generation of CNVs and SDs [49].

Furthermore, we identified a total of 101 human RefSeq-curated genes within the SD blocks. We conducted a Gene Ontology analysis using the ToppFun tool (https://toppgene.cchmc.org, (accessed on 15 October 2023)) with the default parameters. This analysis unveiled that most of the enriched terms were related to the Golgi apparatus (Tables S6 and S7).

Notably, we identified two SDs corresponding to the *GOLGA* and *HERC* gene families mapping within the large SDs (large red and blue arrows) that seem to have a central role in mediating the recurrent microdeletions (Figure 2 and Figure S9).

### 2.4. Segmental Duplication Organization in NHPs

**Figure 3:**
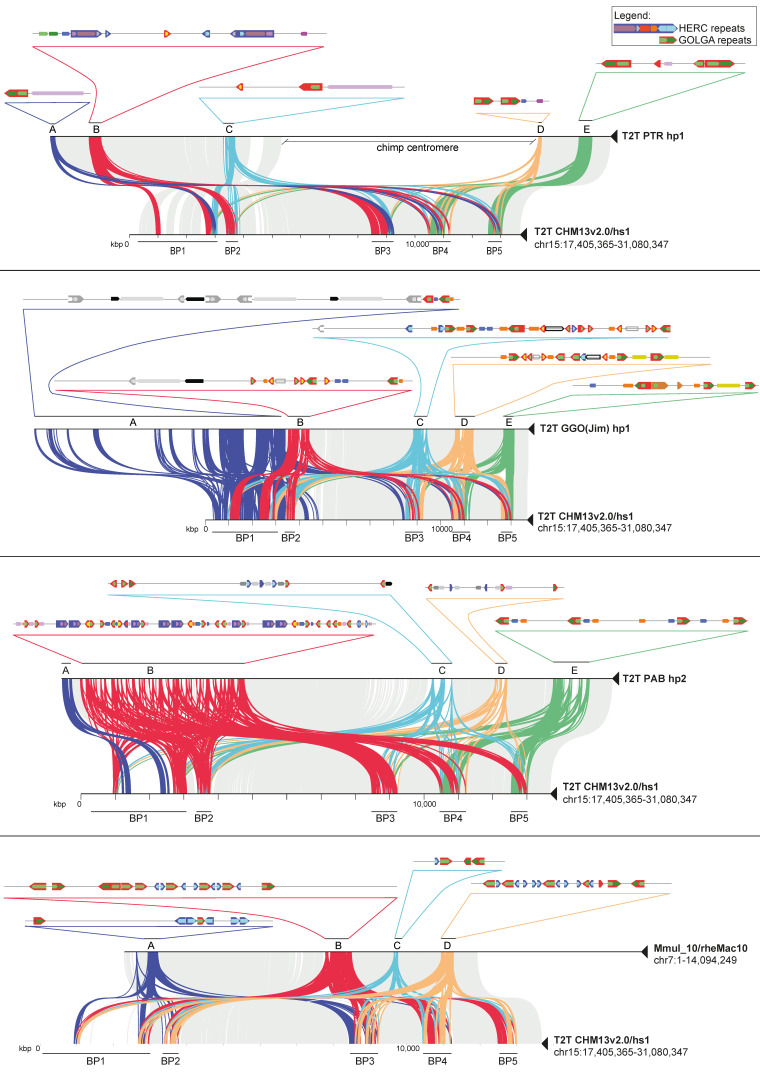
Sequence homology plots of human versus nonhuman primates. Minimiro comparison of the whole 15q11-13 human locus against NHP orthologous regions. Gray lines connect syntenic single-copy regions, while colored lines represent matches between duplication blocks. For each NHP, the duplication blocks are labeled with letters A to E, where a detailed map of the SDs’ organization and their relative orientations are depicted. Larger arrows indicate duplication modules containing core duplicons widespread along the locus.

We tried to estimate the duplication architecture changes during primate evolution comparing the human SDs with the orthologous regions in NHPs. To do this, we took advantage of the macaque reference genome (Mmul_10/rheMac10) (Warren et al., 2020) and the T2T sequence of one chimpanzee, one gorilla, and one orangutan. In order to evaluate the presence of species-specific duplications at the 15q11-q13 region, we performed a minimiro comparison of humans to each NHP, of each primate to itself, and an all-versus-all primate comparison. To avoid confusion with human BPs (BP1, BP2, BP3, BP4, and BP5), we assigned letters to the NHP BPs (BPA, BPB, BPC, BPD, and BPE). Our analysis revealed that a more extensive and complex SD structure has emerged in the evolutionary lineage leading to humans (Figure 2a, Figure 3 and Figure S10) with chimpanzee showing a structure more similar to humans (Figure 3). Nevertheless, the chimpanzee regions syntenic to those involved in human deletions do not have the directly oriented duplication blocks (red and/or blue arrows) that can putatively mediate the same deletions in this species. However, chimpanzee show a ~130 kbp duplication with 97% similarity in direct orientation mapping within BPA and BPC (red–purple arrows), which could mediate the deletion of the syntenic region for human BP2-BP4. Of note, this SD is part of a larger SD (purple–red–blue–red) present in humans and putatively involved in the BP1-BP3 deletion (Figure 2a). All the other chimpanzee BPs do not have directly oriented duplication blocks, other than the *GOLGA* repeats. Interestingly, gorilla is the only NHP enriched in large species-specific duplications (70 to 200 kbp). However, none of these are in direct orientation except for a ~200 kbp SD with 97% similarity mapping at BPA and BPB that could mediate a small deletion (~300 kbp). In addition, a directly oriented ~30 kbp duplication (also present in humans) maps at BPD and BPE (yellow arrows). All the other gorilla BPs do not have directly oriented duplication blocks other than *GOLGA* and *HERC* duplications (Figure 3). Orangutan and macaque BPs are enriched in clusters of short repeats (~20 kbp) corresponding to *GOLGA* and *HERC* (Figure 3). In particular, orangutan shows a burst of duplications mapping at BPB that give the major contribution to the total size (4.2 Mbp out of 4.8 Mbp) of orangutan duplications at this locus. Based on these findings, we can assert that the human lineage exhibits a considerably intricate and extensive SD structure in comparison to non-human primates. This observation suggests that the predisposition to disease-associated large-scale rearrangements has evolved as a distinct characteristic within the human species.

## 3. Discussion

**Figure 4:**
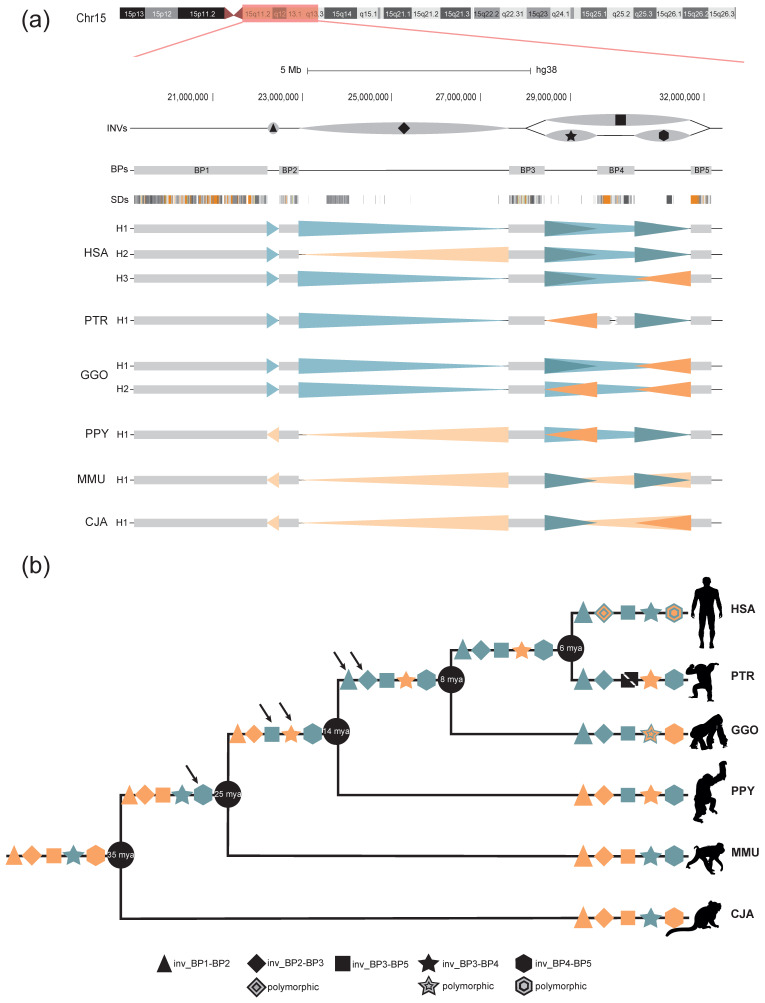
15q11-13 evolutionary history. (**a**) Summary of all the 15q11-q13 haplotypes found in humans and NHPs. Each region is indicated using a different symbol (triangle, rhombus, square, star, and hexagon). Arrowheads indicate the orientation of the region found in this study with teal shades for the direct orientation and orange for the inverted. The human H2 haplotype data are from Porubsky and colleagues [25]. (**b**) Black arrows indicate when the rearrangement occurred during evolution. Teal symbols indicate the direct orientation, orange symbols indicate inversions and patterned symbols polymorphic inversions. Symbols are reported on the branch where the inversion occurred HSA = *Homo sapiens*; PTR *= Pan troglodytes*; GGO *= Gorilla gorilla*; PPY *= Pongo pygmaeus*; MMU *= Macaca mulatta*; CJA = *Callithrix jacchus*.

SDs have historically been difficult to study and resolve using conventional sequencing approaches, making the characterization of variation at these loci extremely challenging. A combination of molecular cytogenetics, sequencing of long molecules, and Strand-seq enabled a new level of comparative genomics analysis in one of the most variable loci of the human genome at 15q11-q13, enriched in large clusters of SDs that render this region genetically unstable. Our study reveals some interesting aspects of the 15q11-q13 genomic instability. During the course of evolution, the region has been independently restructured in different NHP species, resulting in a substantial reorganization primarily due to SD accumulation. Together with these changes, we observed the presence of five large inversions, often shared by different species, that allowed us to distinguish seven different haplotypes in humans and NHPs (Figure 4a). Indeed, two inversions occurred in the African great ape ancestor, two in the great apes ancestor, and one shared by all the Catharrini, with three inversions still polymorphic in at least one species and two events of phylogeny discordance that may be the result of incomplete lineage sorting or recurrent events that have occurred during evolution (Table 1, Figure 4b) [40,50]. Our data confirm the hypothesis that regions flanked by SDs have a significantly higher chance of being recurrent hotspots of rearrangements compared to primate inversions that lack flanking SDs [40].

The comparative analysis of human and NHP SDs at this locus shows a pattern of duplications becoming more complex over the course of evolution with a threefold increased length of duplicated sequences across the 15q11-13 locus in human compared to macaque. However, orangutan represent a notable exception, as they exhibit a burst of SDs at the 15q11-q13 locus. This expansion is centered around the *GOLGA* and *HERC* duplications, confirming the findings already reported by Sudmant and colleagues and Jiang and colleagues [33,51]. Our comparison of human and NHP organization is consistent with the colonization of chromosome 15 by these two duplications around 14–20 million years ago [8,52,53] and it strongly supports the hypothesis that they accumulated their own set of species-specific flanking SDs on the periphery. Thus, we demonstrate that several independent bursts of *GOLGA* and *HERC* duplications, which occurred in the last 15 million years in four different ape lineages, have restructured great ape and human chromosome 15, creating lineage-specific duplication blocks distributed throughout the locus. The complex pattern of human 15q11-q13 exhibits highly identical SDs in both direct and inverted orientation, as opposed to clusters of small inverted SDs organized in tandem as seen in most of the NHP orthologous regions. Notably, most of the large flanking duplications in human are single-copy regions in the NHPs analyzed, suggesting that the core duplicons, with their intrinsic tendency to duplicate, have served to prime large human-specific duplications [33,54]. Similar to other loci [11,55,56], these sequences serve as BPs of numerous disease-causing rearrangements in humans, arising from unequal cross-over events during meiosis, which result in recurrent CNVs.

The significant structural variations in the human genomic organization distinguish it from other primates analyzed, as it is the sole configuration exhibiting large homologous SDs in a direct orientation that flank the disease-critical region between BP1/BP2 and BP3 (Figure S11), implying that susceptibility to large-scale rearrangements associated with disease arose specifically within the human species. Of note, all of the evolutionary inversions we identified and the pathogenic rearrangements described to date at the 15q11-q13 locus [24,57,58,59,60] are linked to the expansion of the *GOLGA* and *HERC* gene families, which are shared between humans and great apes. Our analysis establishes that in three cases out of four (BP2-BP3, BP3-BP5, and BP4-BP5), the inverted orientation is the ancestral state; these include the BP2-BP3 and BP4-BP5 regions, which are the only ones inverted in the human population and they are also hotspots of recurrent CNV associated with disease.

While our study primarily focuses on the evolutionary analysis of structural variations in the 15q11-q13 region, it is worth noting that this region has indeed been recognized as a breakage-prone area, harboring fragile sites within the human genome [61,62,63]. Fragile sites are regions in the genome that are prone to breakage and instability, often resulting in structural variations and genomic rearrangements [64]. The alignment of BPs with these fragile sites suggests a potential connection between the structural evolution of the 15q11-13 locus and genomic fragility. This observation highlights the complex interplay between genomic stability, structural variations, and the evolutionary history of specific genomic regions. It underscores the importance of further research to elucidate the mechanisms and implications of such alignments, which may have significant relevance for understanding the genetic bases of various diseases and disorders.

Structural differences between human haplotypes, based on the literature and current analyses, show the presence of an inversion between BP2 and BP3, previously found at increased rates in mothers of children with the deletion compared to the general population [24,25] (33.2% inverted allele frequency compared to 2.8% in the general population). These findings suggest that the presence of the inversion in a heterozygous state could potentially interfere with the regular process of meiotic synapsis, creating an unusual environment that promotes non-allelic homologous meiotic recombination between SDs. However, the inversion does not appear to be a prerequisite for pathogenic rearrangement formation since the direct haplotype could also undergo this process. Previous studies have identified an increasing number of inversions associated with susceptibilities to recurrent genomic rearrangements [5,11,25,55,56]. In certain instances, inversions of these regions seem to be a prerequisite for subsequent rearrangement to take place [65]. An example is the 17q21.31 locus, where specific duplications arose in direct orientation, predisposing the H2 haplotype to disease-associated rearrangements. In all patients investigated thus far, the 17q21.31 microdeletion occurs exclusively in chromosomes harboring the inversion of that specific region. This observation strongly suggests that inversions play a role in establishing a particular genomic configuration that is required for the occurrence of abnormal meiotic rearrangements at these loci. While inversions in the 15q11-q13 region are observed at increased rates compared to the general population, they do not appear to be a prerequisite for illegitimate meiotic recombination since the direct haplotype can also undergo the recurrent copy number variation associated with disease. Our detailed analysis of the direct T2T sequenced haplotype (T2T CHM13v2.0/hs1) identified the presence of four pairs of duplication blocks in direct orientation carrying *GOLGA* and *HERC* duplications that likely promote non-allelic homologous recombination, leading to rearrangement and disease (Figure 2). Indeed, the duplication of the cores flanking the critical region resulted in a 16-fold expansion in human when compared to macaque of the size of flanking, high-identity (98–99%), directly oriented SDs, theoretically predisposing the locus to recurrent copy number variation via unequal crossover specifically in the human lineage. Our findings emphasize the impact of SDs and the associated genetic variation with respect to human health and genomic susceptibility to disease. We show that the human architecture has evolved more extensive sequence homology compared to other apes, perhaps explaining its tendency to undergo recurrent rearrangements associated with disease. The occurrence of *GOLGA* core duplicons at numerous disease-associated rearrangements and evolutionary BPs supports the hypothesis that *GOLGA* repeats have played a fundamental role in shaping the chromosome 15 architecture in both humans and great apes. Their highly mutagenic nature predisposes our genome to DNA breakage with consequences for human disease and evolution [8,10]. Future in-depth analyses of inverted haplotypes and the BP resolution of patient chromosomes might unveil genomic features that may increase mutability in the population and predispose inversion carriers to de novo disease-causing CNVs.

## 4. Materials and Methods

### 4.1. Primate Genome Sequence Data

Contiguous genome sequence assemblies from the Telomere-to-Telomere consortium were obtained from male chimpanzee (*Pan troglodytes*), Western gorilla (*Gorilla gorilla*), and Bornean orangutan (*Pongo pygmaeus*) and downloaded from GenomeArk (Data Availability Statement). We subsetted whole-genome assemblies to our region of interest (chr15:17405365-31080347). For this purpose, we first aligned the above-mentioned whole-genome assemblies with the T2T-CHM13 (v2.0) reference using the “asm-to-reference-alignment” pipeline (https://github.com/mrvollger/asm-to-reference-alignment/, (accessed on 31 May 2023)). Next, we used rustybam (version 0.1.27, zenodo doi: 10.5281/zenodo.5875012) to subset the resultant PAF alignments only to the above-mentioned region of interest and extracted the FASTA sequences using R packages Biostrings and GenomicRanges [66,67]. The extracted FASTA files were then further analyzed as outlined below.

### 4.2. FISH Analysis

The interphase nuclei were obtained from lymphoblast and fibroblast cell lines from 13 human HapMap individuals (Coriell Cell Repository, Camden, NJ, USA), 5 chimpanzees (*Pan troglodytes*), 4 gorillas (*Gorilla gorilla*), 5 orangutans (*Pongo pygmaeus*), 3 macaques (*Macaca mulatta* and *Macaca fascicularis*), and 1 marmoset (*Callithrix jacchus*) available at the University of Bari (Table S4). The FISH experiments were performed using human fosmid (n = 7) and BAC (n = 3) clones; the anchor probes were changed when necessary, depending on the species-specific locus organization (Table S4). Clones were directly labeled using nick translation with Cy3-dUTP (PerkinElmer), Cy5-dUTP (PerkinElmer), and fluorescein-dUTP (Enzo) as described by Lichter et al. [68], with minor modifications. Briefly, 300 ng of labeled probe was used for the FISH experiments; hybridization was performed at 37 °C in 2xSSC, 50% (*v*/*v*) formamide, 10% (*w*/*v*) dextran sulfate, and 3 mg sonicated salmon sperm DNA, with a volume of 10 mL. Posthybridization washing was carried out at 60 °C in 0.1xSSC (three times, high stringency, for hybridizations on human, chimpanzee, gorilla, and orangutan) or at 37 °C in 2xSSC and 42 °C in 2xSSC, 50% formamide (three times each, low stringency, for hybridizations on macaque). The nuclei were simultaneously DAPI-stained. Digital images were obtained using a Leica DMRXA2 epifluorescence microscope equipped with a cooled CCD camera (Princeton Instruments). The DAPI, Cy3, Cy5, and fluorescein fluorescence signals, detected with specific filters, were recorded separately as grayscale images. Pseudocoloring and merging of the images were performed using the Adobe Photoshop software. Proximal and distal inversions were interrogated using two probes within the putative inversion region and a reference probe outside, as previously described [9].

### 4.3. Segmental Duplication Detection

The SD content of human and NHPs was assessed using the T2T sequences for human (CHM13v2.0/hs1), chimpanzee, gorilla, and orangutan and the genome reference for macaque (Mmul_10/rheMac10). High-identity sequence alignments were generated using a modified version of Miropeats [69] that uses minimap2 to identify alignments. The code for the homology plots can be found at https://github.com/mrvollger/assembly_workflows (accessed on 15 April 2023) under workflows/minimiro.smk [70], as described before [18]. In brief, the sequences are aligned using the following minimap2 parameters: asm5 for human, chimpanzee, gorilla, orangutan, and macaque versus themselves and human versus chimpanzee, gorilla, and orangutan, and asm20 for human versus macaque; for all hominoids, the sequence identity alignment thresholds were set at s500, with the exception of orangutan and macaque, where a s200 threshold was used to capture more divergent macaque sequence reads aligned with the human genome. Each human and NHP BP was analyzed two at time, processed into a postscript file using scripts/minimiro.py, and converted into a PDF.

## Figures and Tables

**Table 1:** 15q11-q13 inversion status and evolution in human and NHPs.

Species	BP1-BP2 Inversion chr15:20454276-21173858	BP2-BP3 Inversion chr15:21173858-25860219	BP3-BP5 Inversion chr15:26608046-29923396	BP3-BP4 Inversion chr15:26617330-27847082	BP4-BP5 Inversion chr15:28677026-29923396
*Homo sapiens*	dir/dir	dir/inv	dir/dir	dir/dir	dir/inv
*Pan troglodytes*	dir/dir	dir/dir	n.a. *	inv/inv	dir/dir
*Gorilla gorilla*	dir/dir	dir/dir	dir/dir	dir/inv	inv/inv
*Pongo pygmaeus*	inv/inv	inv/inv	dir/dir	inv/inv	dir/dir
*Macaca mulatta*	inv/inv	inv/inv	inv/inv	dir/dir	dir/dir
*Callithrix jacchus* (outgroup)	inv/inv	inv/inv	inv/inv	dir/dir	inv/inv
**Evolution**	African great ape ancestor	African great ape ancestor	Great apes ancestor	Great apes ancestor	Catharrini ancestor

* not tested because the region is interrupted by a *Pan*-specific inversion. dir = direct orientation; inv = inverted orientation. Coordinates are based on the T2T CHM13v2.0/hs1 reference. Coordinates are based on the T2T CHM13v2.0/hs1 reference.

## Data Availability

The T2T assemblies of three NHP genomes (chimpanzee, gorilla, and orangutan) were obtained from the GenomeArk repository (https://genomeark.github.io/t2t-all/, (accessed on 31 May 2023)). All long-read data and assemblies were generated by the members of the Telomere-to-Telomere consortium. The assemblies of our selected region (for the above-mentioned NHP genomes) have been made available as a courtesy of the T2T Primates Consortium.

## Supplementary Materials

The following supporting information can be downloaded at https://www.mdpi.com/article/10.3390/ijms242115818/s1.
