# Supplementary material for: Structural Variation Evolution at the 15q11-q13 Disease-Associated Locus
Source: Int J Mol Sci. 2023 Oct 31;24(21):15818. doi: 10.3390/ijms242115818 (PMC10648317; doi:10.3390/ijms242115818)
Supplement: Supplementary file 1 [file ijms-24-15818-s001.zip › FigureS1.pdf]

Figure S1

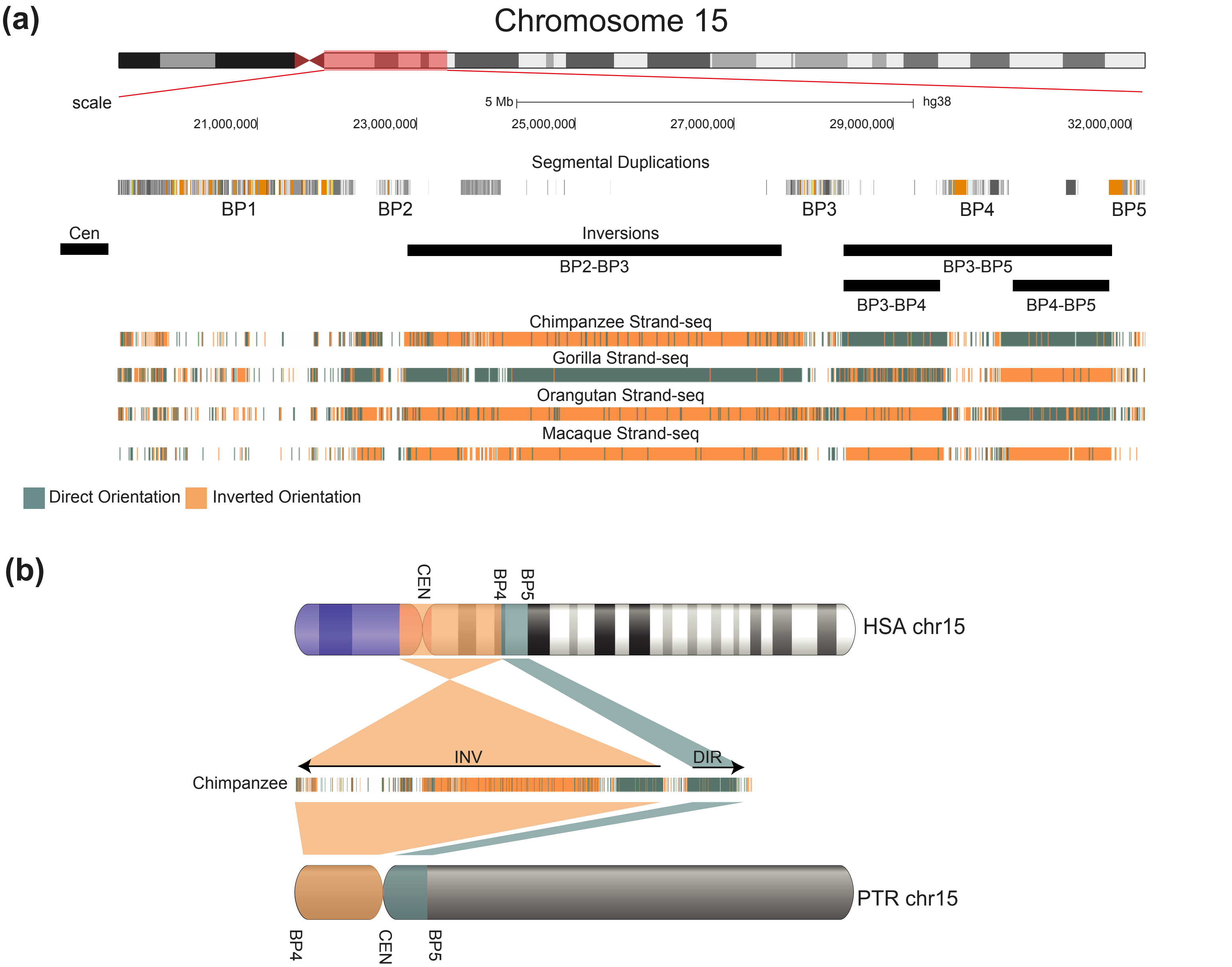

Figure S1: Strand-seq analysis of the 15q11-13 locus in primates. (a) UCSC Genome Browser view (GRCh38/hg38) of the whole locus in human. Black bars represent the four classes of inver-sions. Strand-seq data for chimpanzee, gorilla, orangutan, and macaque are reported with teal reads representing the direct orientation with respect to human, while orange reads indicate the inverted orientation. **(b)** Human and chimpanzee chromosome 15 ideograms together with chim-panzee Strand-seq are shown. The image shows the Pan-specific pericentric inversion between centromere and BP4; thus, the orientation of the cen-BP4 region based on Strand-seq appears op-posite to its actual orientation.
