# Supplementary material for: Structural Variation Evolution at the 15q11-q13 Disease-Associated Locus
Source: Int J Mol Sci. 2023 Oct 31;24(21):15818. doi: 10.3390/ijms242115818 (PMC10648317; doi:10.3390/ijms242115818)

Figure S10

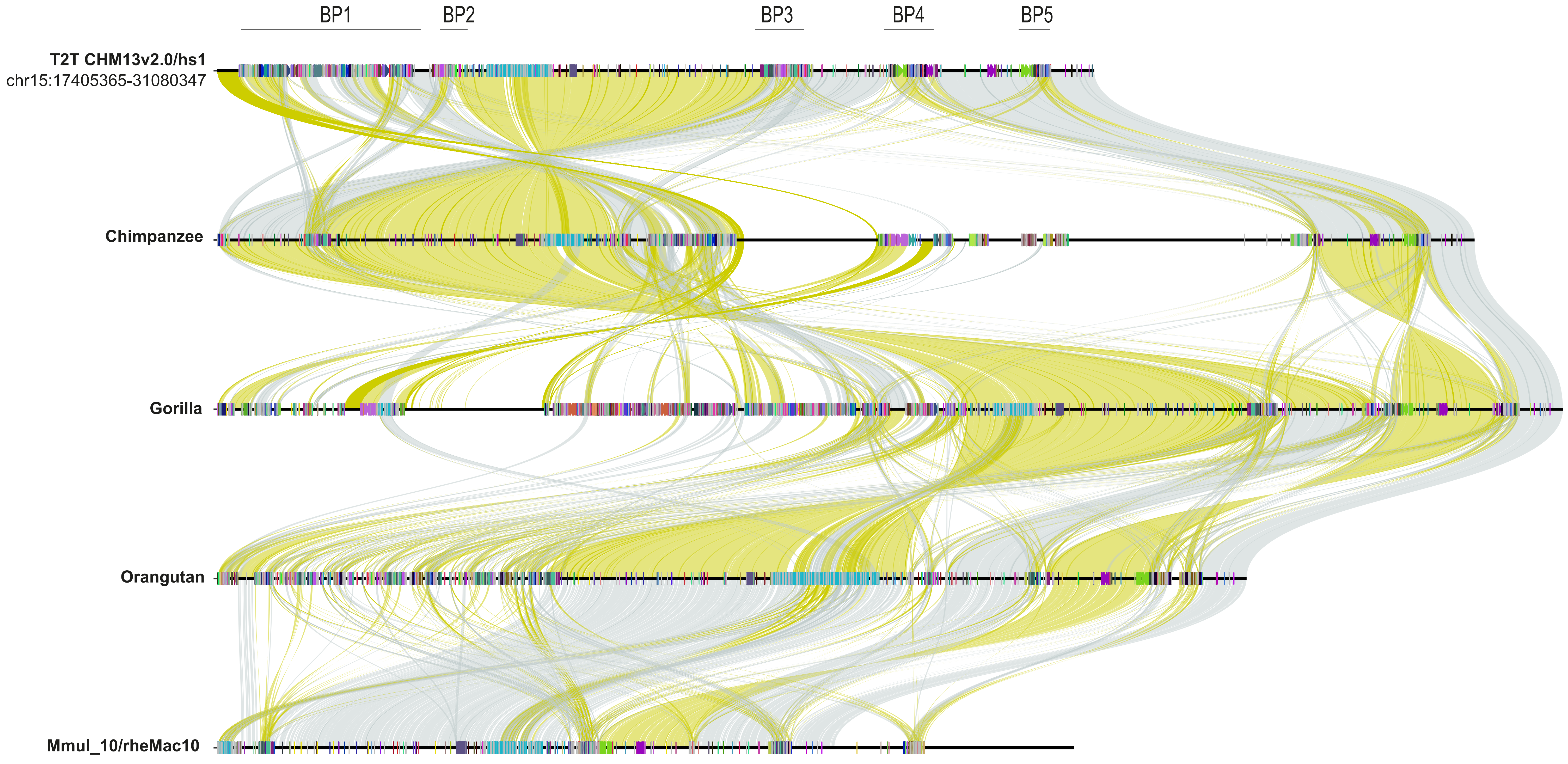

Figure S10. All-versus-all primate sequence homology plots. Minimiro comparison of the whole 15q11-13 human locus and the NHP orthologous regions.Yellow lines represent sequences showing a relative inverted orientation, while grey lines represent sequences showing a relative direct orientation. Colored boxes indicate SDs.

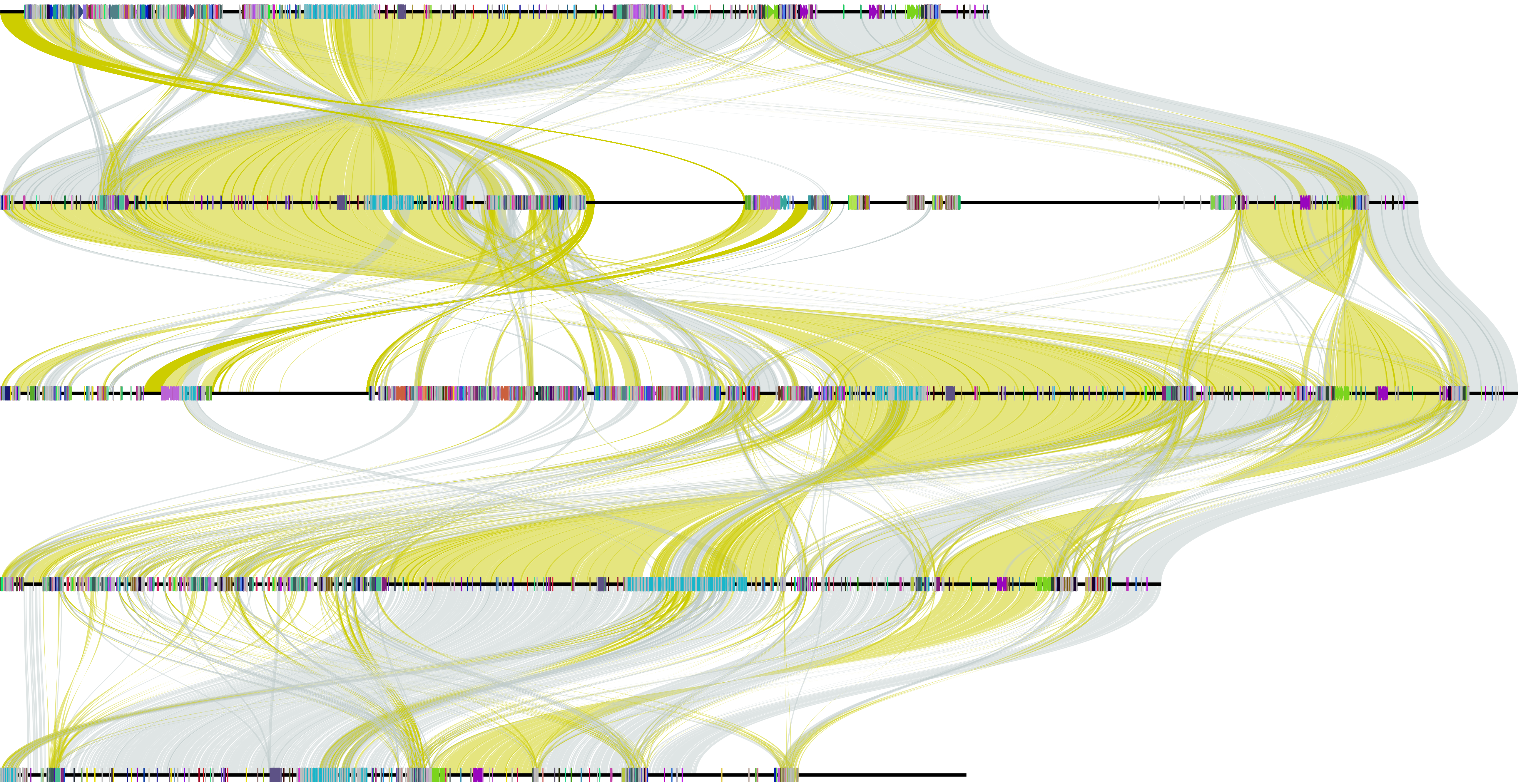

Supplement: Supplementary file 1 [file ijms-24-15818-s001.zip › FigureS10.pdf]
