# Supplementary material for: Structural Variation Evolution at the 15q11-q13 Disease-Associated Locus
Source: Int J Mol Sci. 2023 Oct 31;24(21):15818. doi: 10.3390/ijms242115818 (PMC10648317; doi:10.3390/ijms242115818)
Supplement: Supplementary file 1 [file ijms-24-15818-s001.zip › FigureS11.pdf]

Figure S11

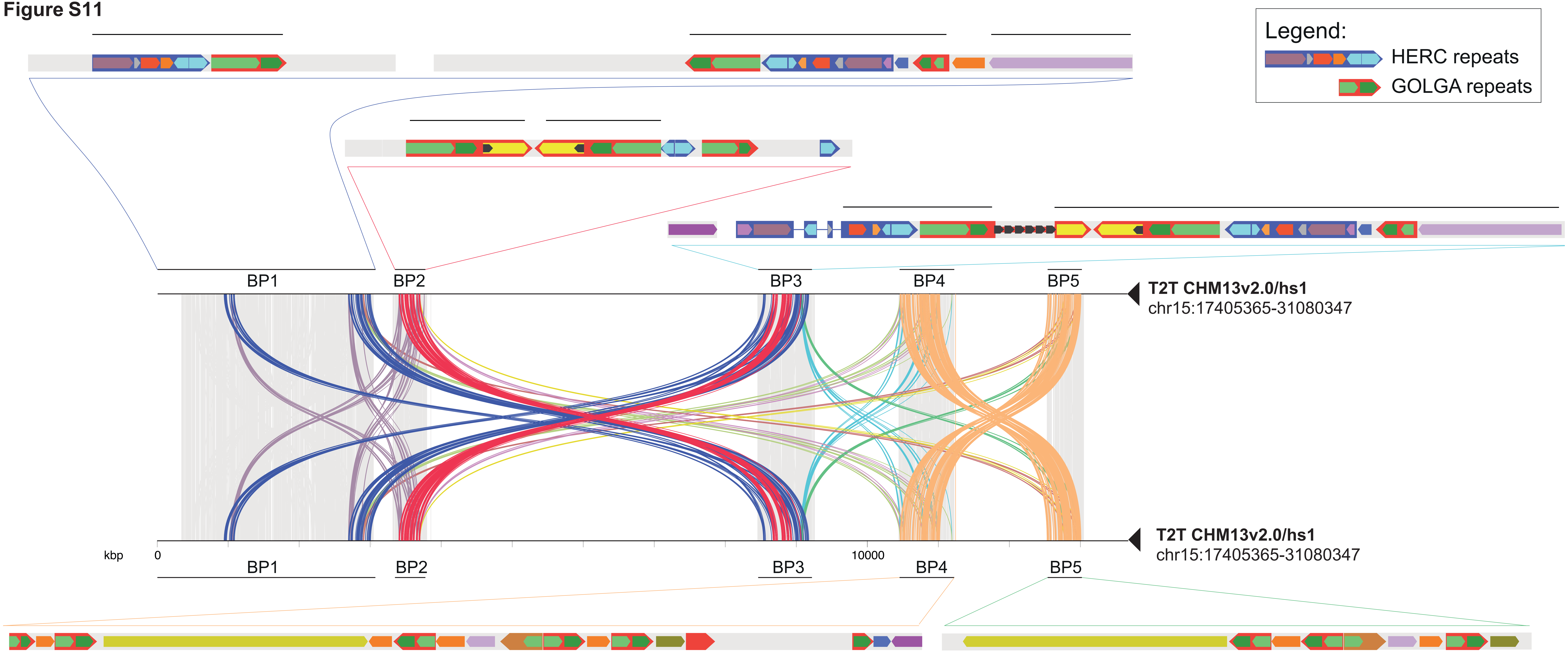

Figure S11:Human-specific directly oriented duplications. Minimiro comparison of the whole 15q11-13 human locus against itself (T2T CHM13v2.0/hs1). The five SD blocks involved in pathogenic rearrangements are depicted (BP1 to BP5). Colored lines connect paralogous SDs between different BPs. A detailed map of the SDs organization and their relative orientation is depicted for each BP. Larger arrows indicate duplication modules containing core duplicons widespread along the locus. Black lines above the arrows indicate human-specific duplications in direct orientation.
