# Supplementary material for: Structural Variation Evolution at the 15q11-q13 Disease-Associated Locus
Source: Int J Mol Sci. 2023 Oct 31;24(21):15818. doi: 10.3390/ijms242115818 (PMC10648317; doi:10.3390/ijms242115818)
Supplement: Supplementary file 1 [file ijms-24-15818-s001.zip › FigureS2.pdf]

Figure S2

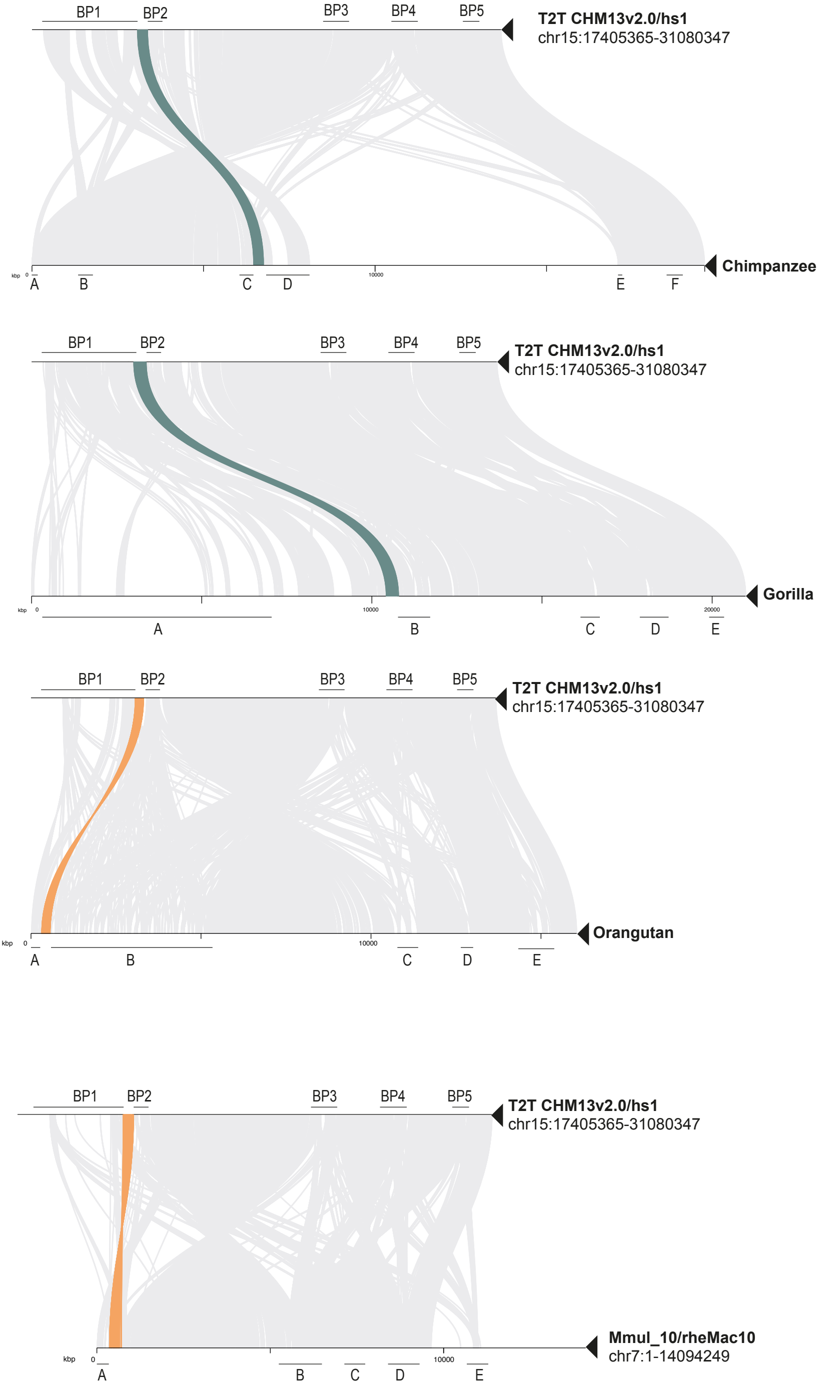

Figure S2: BP1-BP2 sequence homology plots. Minimiro sequence homology plots between hu-man and NHPs show that the BP1-BP2 region is inverted in orangutan and macaque, while chim-panzee and gorilla have the same orientation as human. Teal and orange lines represent the BP1-BP2 region in direct and inverted orientation between humans and NHPs, respectively. Gray lines connect all the 15q11-13 regions not involved with the BP1-BP2 single-copy region.
