# Supplementary material for: Structural Variation Evolution at the 15q11-q13 Disease-Associated Locus
Source: Int J Mol Sci. 2023 Oct 31;24(21):15818. doi: 10.3390/ijms242115818 (PMC10648317; doi:10.3390/ijms242115818)
Supplement: Supplementary file 1 [file ijms-24-15818-s001.zip › FigureS3.pdf]

Figure S3

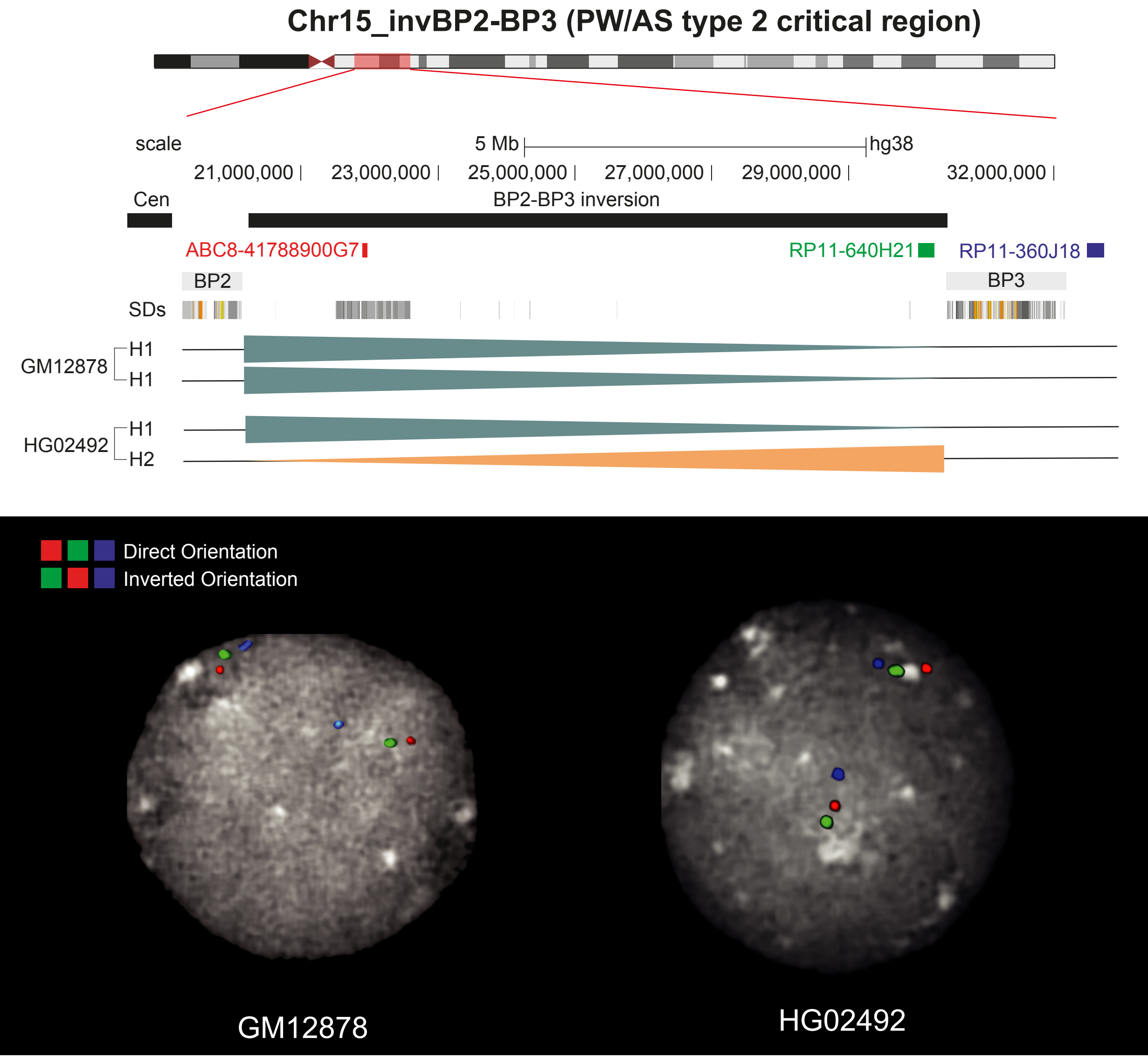

Figure S3: FISH validation of the BP2-BP3 human polymorphic inversion. UCSC Genome Browser view (GRCh38/hg38) of the BP2-BP3 region at the 15q11-13 locus. The tested inversion is shown with a black bar and fosmid and BAC clones used for FISH experiments are indicated with red, green, and blue colored blocks. The two human haplotypes (H1 and H2) for the two tested individuals are shown as teal and orange arrowheads respectively. FISH results are shown in the bottom of the panel. The color order indicates probes relative orientation, with red-green-blue signals showing haplotypes in direct orientation and green-red-blue in inverted haplotypes. FISH analyses of the inversion status show that GM12878 is homozygous for the direct haplotype while HG02492 is heterozygous for the BP2-BP3 inversion.
