# Supplementary material for: Structural Variation Evolution at the 15q11-q13 Disease-Associated Locus
Source: Int J Mol Sci. 2023 Oct 31;24(21):15818. doi: 10.3390/ijms242115818 (PMC10648317; doi:10.3390/ijms242115818)
Supplement: Supplementary file 1 [file ijms-24-15818-s001.zip › FigureS4.pdf]

Figure S4

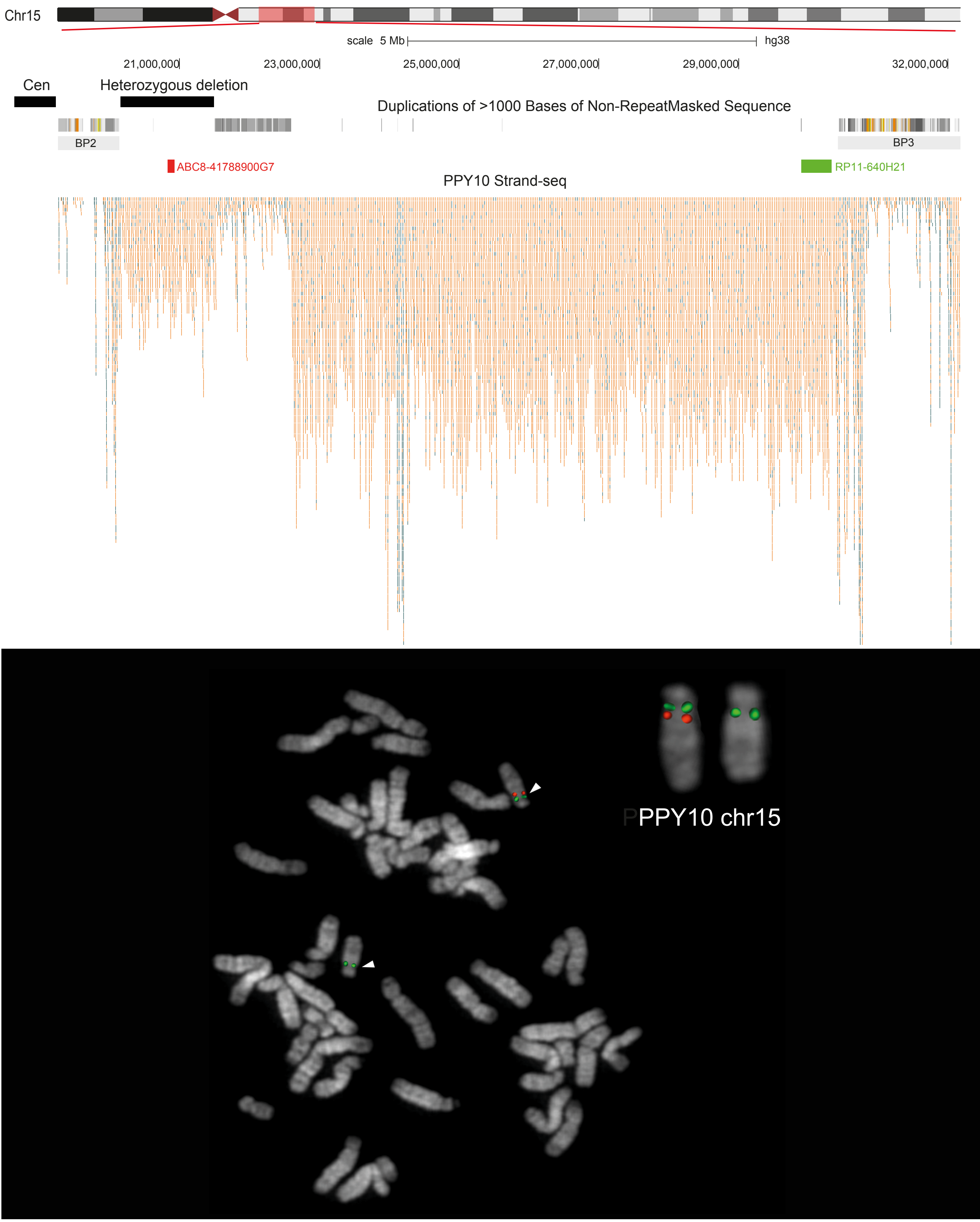

Figure S4: Orangutan heterozygous deletion. The top panel shows a UCSC Genome Browser view (GRCh38/hg38) of the human BP2-BP3 region. A squished view of orangutan (PPY10) Strand-seq data is shown. Reads mapping within the region indicated with a black bar show a read depth decrease indicating the presence of a deletion in heterozygous state. FISH validation of the PPY10 deletion is shown in the bottom panel.
