# Supplementary material for: Structural Variation Evolution at the 15q11-q13 Disease-Associated Locus
Source: Int J Mol Sci. 2023 Oct 31;24(21):15818. doi: 10.3390/ijms242115818 (PMC10648317; doi:10.3390/ijms242115818)
Supplement: Supplementary file 1 [file ijms-24-15818-s001.zip › FigureS5.pdf]

Figure S5

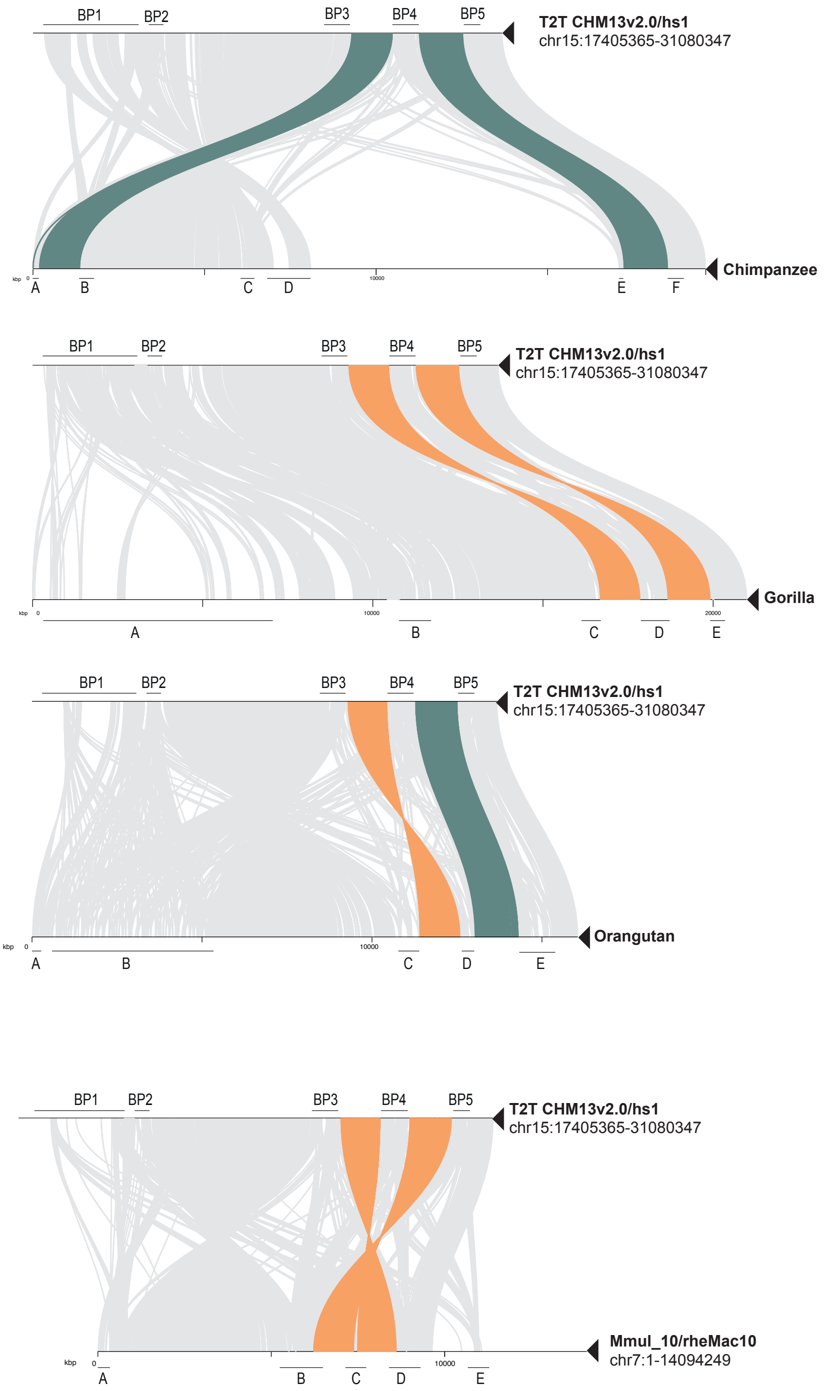

Figure S5: S5. BP3-BP4 and BP4-BP5 sequence homology plots. Minimiro sequence homology plots between human and NHPs show that the BP3-BP4 and BP4-BP5 regions occurred as two separate inversions in all the analyzed NHPs with the exception of macaque. Teal and orange lines represent the two regions in direct and inverted orientation between humans and NHPs, respec-tively. Gray lines connect all the regions non-homologous to BP3-BP4 and BP4-BP5.
