# Supplementary material for: Structural Variation Evolution at the 15q11-q13 Disease-Associated Locus
Source: Int J Mol Sci. 2023 Oct 31;24(21):15818. doi: 10.3390/ijms242115818 (PMC10648317; doi:10.3390/ijms242115818)
Supplement: Supplementary file 1 [file ijms-24-15818-s001.zip › FigureS6.pdf]

Figure S6

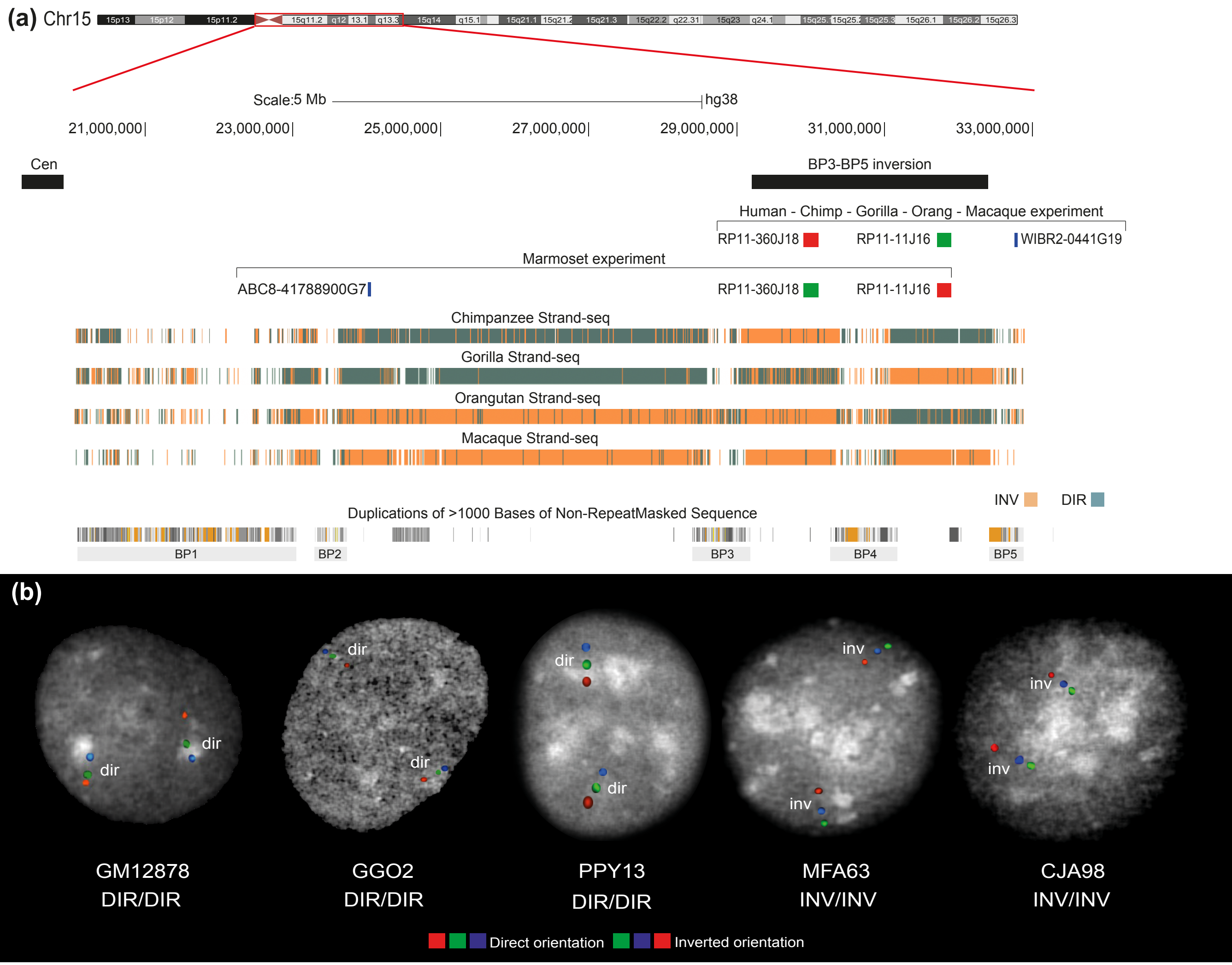

Figure S6: BP3-BP5 inversion analysis. (a) UCSC Genome Browser view (GRCh38/hg38) of the BP3-BP5 region in human. The black bar represents the putative inversion. Fosmid and BAC clones used for FISH experiments on interphase nuclei are indicated with black blocks. Strand-seq data for chimpanzee, gorilla, orangutan, and macaque are reported. **(b)** FISH results on interphase nuclei for the BP3-BP5 inversion in each analyzed species. The color order indicates the probe's relative orientation, with red-green-blue signals showing haplotypes in direct orientation and green-red-blue signals showing inverted haplotypes. FISH analyses show that macaque and mar-moset (outgroup) are inverted when compared to the human reference genome orientation, while chimpanzee, gorilla, and orangutan are in direct orientation. GM12878 = *Homo sapiens*; PTR = *Pan troglodytes*; GGO = *Gorilla gorilla*; PPY = *Pongo pygmaeus*; MFA = *Macaca fascicularis*; CJA = *Callithrix jacchus*.
