# Supplementary material for: Structural Variation Evolution at the 15q11-q13 Disease-Associated Locus
Source: Int J Mol Sci. 2023 Oct 31;24(21):15818. doi: 10.3390/ijms242115818 (PMC10648317; doi:10.3390/ijms242115818)
Supplement: Supplementary file 1 [file ijms-24-15818-s001.zip › FigureS8.pdf]

Figure S8

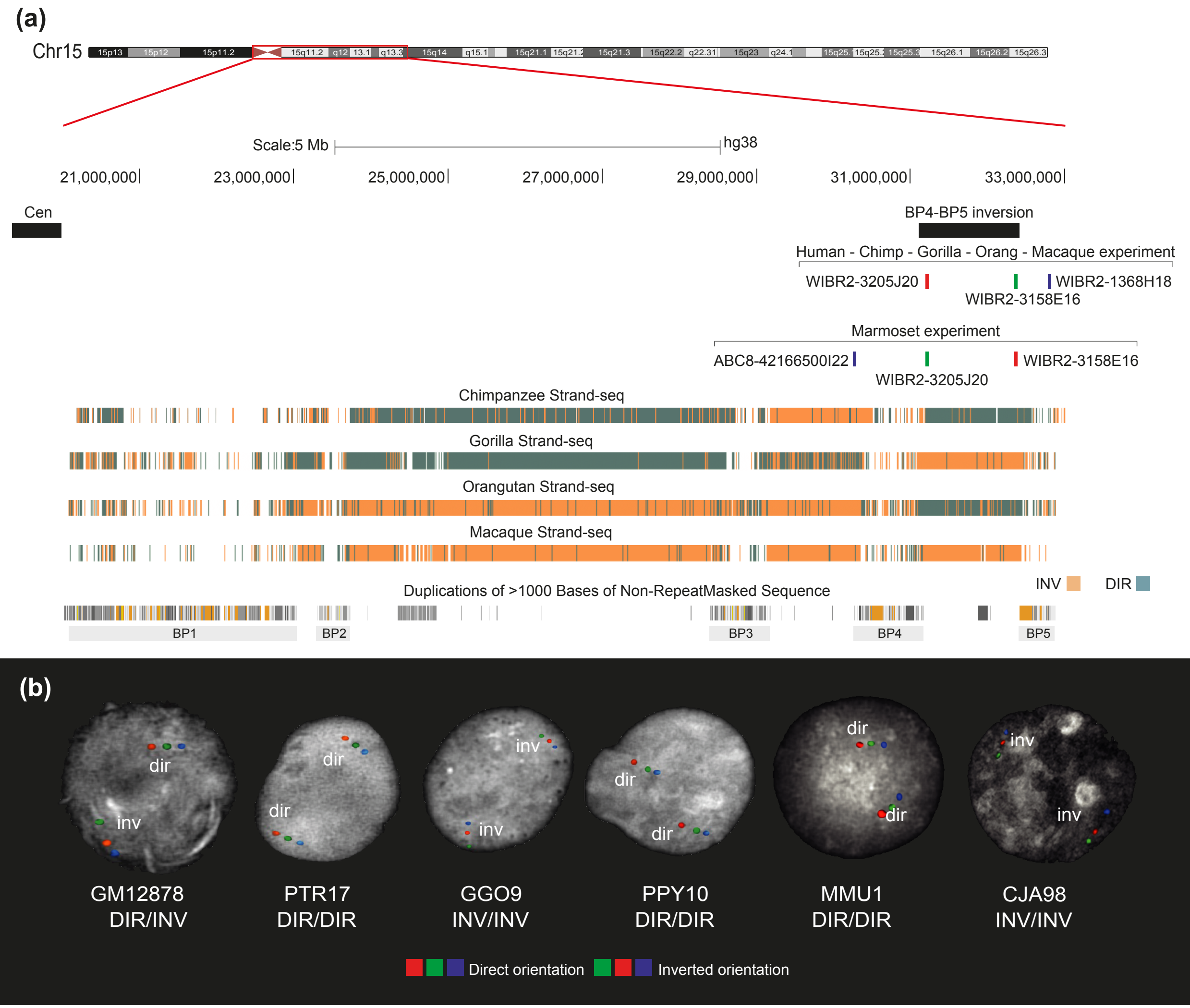

Figure S8: BP4-BP5 inversion analysis. (a) UCSC Genome Browser view of the BP4-BP5 region in human. The black bar represents the putative inversion and fosmid and BAC clones used for FISH experiments on interphase nuclei are indicated with black blocks following their names. Strand-seq data for chimpanzee, gorilla, orangutan, and macaque are reported showing an in-verted orientation for gorilla and macaque and a direct orientation for chimpanzee and orangutan. **(b)** FISH results on interphase nuclei for the BP4-BP5 inversion in each analyzed species are shown. The color order indicates probes relative orientation, with red-green-blue signals showing haplotypes in direct orientation and green-red-blue signals showing inverted haplotypes. FISH analyses show that gorilla and marmoset are inverted when compared to the human reference genome orientation, while chimpanzee, orangutan, and macaque are in direct orientation. The human used as a control shows a heterozygous state of the inversion. GM12878 = *Homo sapiens*; PTR = *Pan troglodytes*; GGO = *Gorilla gorilla*; PPY = *Pongo pygmaeus*; MMU = *Macaca mulatta*; CJA = *Callithrix jacchus*.
