# Supplementary material for: Structural Variation Evolution at the 15q11-q13 Disease-Associated Locus
Source: Int J Mol Sci. 2023 Oct 31;24(21):15818. doi: 10.3390/ijms242115818 (PMC10648317; doi:10.3390/ijms242115818)
Supplement: Supplementary file 1 [file ijms-24-15818-s001.zip › FigureS9.pdf]

Figure S9

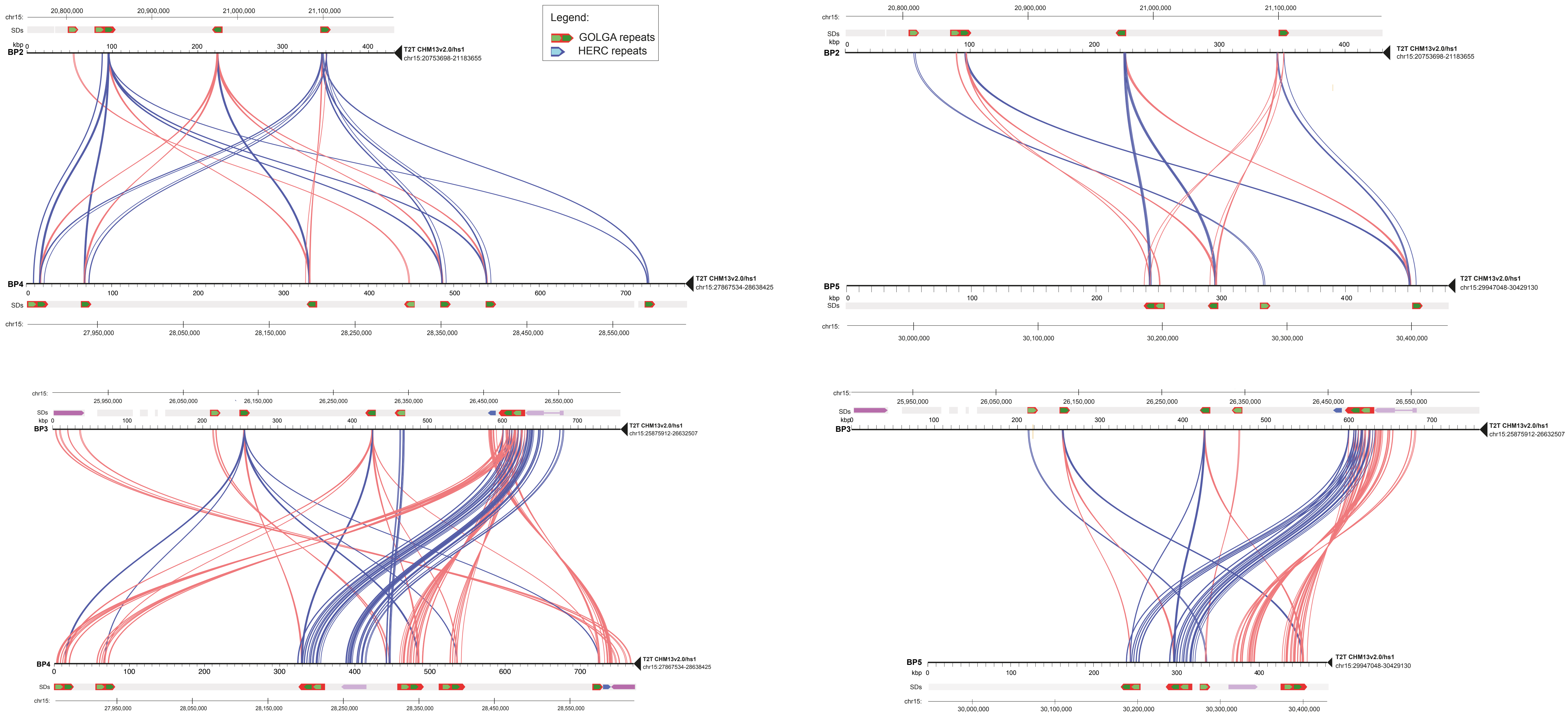

Figure S9: Human sequence homology plots of BPs not involved in recurrent CNVs. Minimiro comparison of BP2 versus BP4, BP2 versus BP5, BP3 versus BP4, and BP3 versus BP5 highlight homologous SDs between the BPs. Red lines represent sequences showing a relative inverted ori-entation between the two BPs, while blue lines represent sequences showing a relative direct ori-entation.
