# Supplementary material for: Structural Variation Evolution at the 15q11-q13 Disease-Associated Locus
Source: Int J Mol Sci. 2023 Oct 31;24(21):15818. doi: 10.3390/ijms242115818 (PMC10648317; doi:10.3390/ijms242115818)
Supplement: Supplementary file 1 [file FigureS7.pdf › FigureS7.pdf]

Figure S7

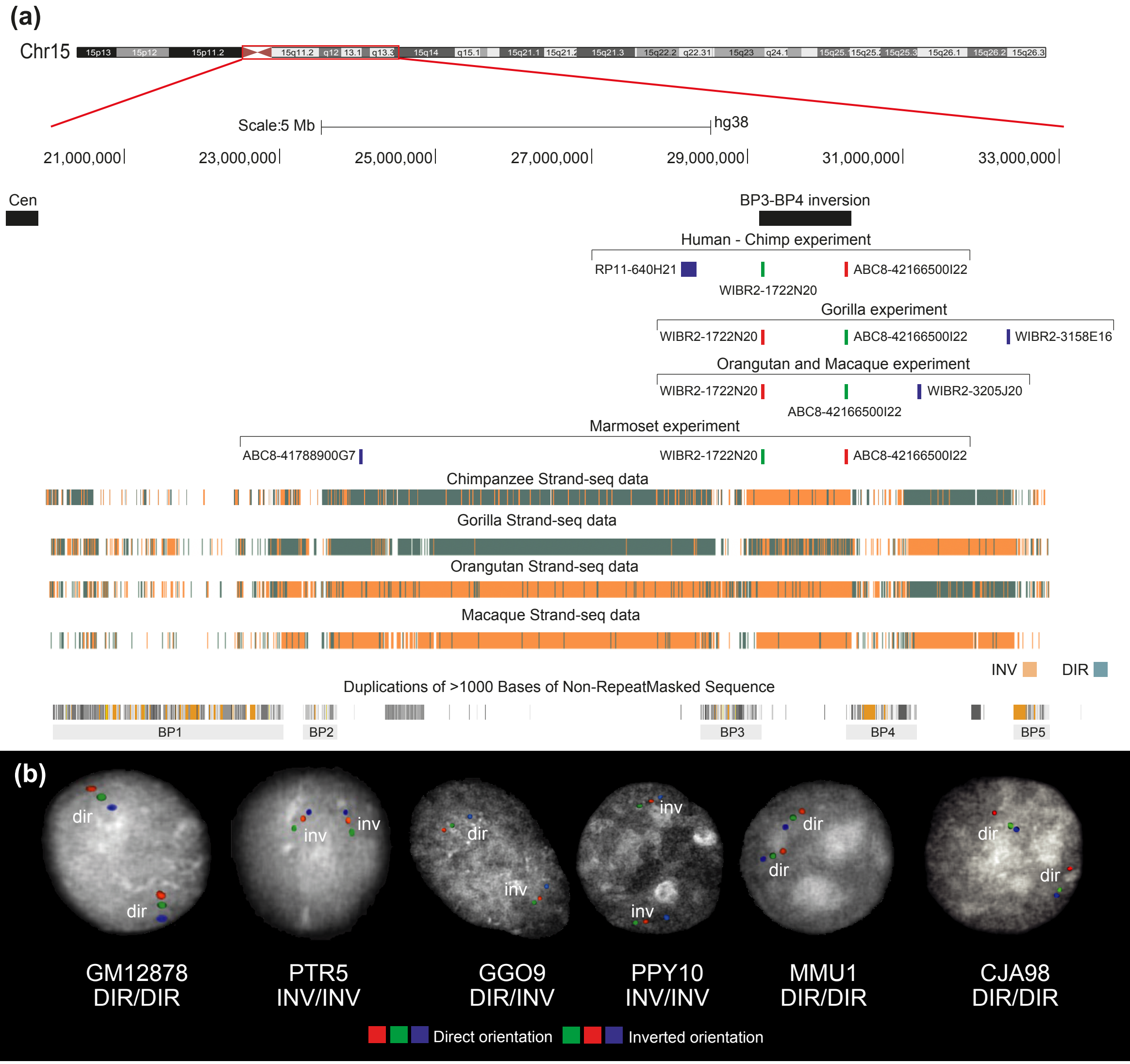

Figure S7: BP3-BP4 inversion analysis. (a) UCSC Genome Browser view of the BP3-BP4 region in human. The black bar represents the putative inversion and fosmid and BAC clones used for FISH experi-ments on interphase nuclei are indicated with black blocks following their names. Strand-seq data for chimpanzee, gorilla, orangutan, and macaque are reported showing an in-verted orientation for all the species with the exception of gorilla that shows a heterozygous state of the region. **(b)** FISH results on interphase nuclei for the BP3-BP4 inversion in each analyzed species are shown. The color order indicates probes relative orientation, with red-green-blue sig-nals showing haplotypes in direct orientation and green-red-blue signals showing inverted hap-lotypes. FISH analyses show that orangutan and chimpanzee are inverted when compared to hu-man, gorilla is heterozygous for the region, and macaque and marmoset (outgroup) are in direct orientation. GM12878 = *Homo sapiens*; PTR = *Pan troglodytes*; GGO = *Gorilla gorilla*; PPY = *Pongo pygmaeus*; MMU = *Macaca mulatta*; CJA = *Callithrix jacchus*.
